# Vertical Corner Feature Based Precise Vehicle Localization Using 3D LIDAR in Urban Area

**DOI:** 10.3390/s16081268

**Published:** 2016-08-10

**Authors:** Jun-Hyuck Im, Sung-Hyuck Im, Gyu-In Jee

**Affiliations:** 1 Department of Electronic Engineering, Konkuk University, 120 Neungdong-ro, Gwangjin-gu, Seoul 05029, Korea; junhyuck@konkuk.ac.kr; 2 Satellite Navigation Team, Korea Aerospace Research Institute, 169-84 Gwahak-ro, Yuseong-gu, Daejeon 305-806, Korea; ish@kari.re.kr

**Keywords:** precise vehicle localization, vertical corner feature, urban area, corner map, 3D LIDAR

## Abstract

Tall buildings are concentrated in urban areas. The outer walls of buildings are vertically erected to the ground and almost flat. Therefore, the vertical corners that meet the vertical planes are present everywhere in urban areas. These corners act as convenient landmarks, which can be extracted by using the light detection and ranging (LIDAR) sensor. A vertical corner feature based precise vehicle localization method is proposed in this paper and implemented using 3D LIDAR (Velodyne HDL-32E). The vehicle motion is predicted by accumulating the pose increment output from the iterative closest point (ICP) algorithm based on the geometric relations between the scan data of the 3D LIDAR. The vertical corner is extracted using the proposed corner extraction method. The vehicle position is then corrected by matching the prebuilt corner map with the extracted corner. The experiment was carried out in the Gangnam area of Seoul, South Korea. In the experimental results, the maximum horizontal position error is about 0.46 m and the 2D Root Mean Square (RMS) horizontal error is about 0.138 m.

## 1. Introduction

Precise vehicle localization has become important for the safe driving of autonomous vehicles. Generally, a Global Positioning System (GPS) is most often used for position recognition. However, the position accuracy of the GPS is not reliable in urban areas. To solve this problem, techniques for recognizing position by integrating several sensors (e.g., GPS, Inertial Measurement Unit (IMU), Vision, light detection and ranging (LIDAR), etc.) have been continuously researched. Kummerle et al. [1] presented an autonomous driving system in a multi-level parking structure. This approach utilizes multi-level surface maps to localize a self-driving car based on 3D LIDAR. Brenner [2] presented a vehicle localization approach using the typical roadside pole obtained by 2D LIDAR. Baldwin and Newman [3] and Chong et al. [4] presented a vehicle localization method with 2D push-broom LIDAR and 3D priors. Particularly, [4] utilized LIDAR accumulation with a 3D rolling window. Hu et al. [5], Choi and Maurer [6,7] presented hybrid map-based localization methods. The topological-metric hybrid map of [5] include the lane marking and 2D occupancy grid map. Also, the grid-feature hybrid map of [6,7] includes the typical roadside pole and 2D occupancy grid map. Thus, many methods have been researched for vehicle localization. However, these methods did not provide a complete solution for an urban area.

Recently, a localization method using a Velodyne LIDAR based road intensity map has been researched. Levinson et al. [8,9], Hata and Wolf [10] presented an intensity map based localization method using the Velodyne LIDAR. Wolcott and Eustice [11] presented a visual localization method within a LIDAR intensity map. The LIDAR based road intensity map matching method has the advantage of a very high localization accuracy. The camera based road intensity map matching method has the advantage of being able to be used with an already mounted low cost camera. However, both methods have the disadvantage of not being able to be used when the vehicle is in congested traffic in an urban area. Also, the data file size of the road intensity map is very large. Most recently, Wolcott and Eustice [12] presented a new scan matching algorithm that leverages Gaussian mixture maps. This solution is able to overcome the lack (harsh weather and poor road surface texture) of a road intensity map by exploiting the structure.

Because tall buildings in urban areas obstruct the GPS satellite signal reception, the position accuracy of the GPS is very poor. However, buildings in urban areas can be used to generate a landmark map for precise vehicle localization. This map’s information can be represented in various forms (e.g., 2D/3D occupancy grid map, 3D point cloud, 2D corner feature, 2D contour (line), and 3D vertical surface, etc.). We focused on the outer wall of a building which is erected vertically to the ground and almost flat. These corners can provide very good landmarks and also can be extracted using the LIDAR. In contrast to the use of road marking, corners can be extracted during traffic congestion periods.

A significant amount of research has been carried out on the corner detection method using LIDAR [13,14,15,16,17]. In addition, a large number of papers have been presented on the position estimation of a mobile robot using detected corner points. However, most of these papers focused on the indoor localization of the mobile robot [18,19,20,21] and research on vehicle localization using the vertical corner extraction based on LIDAR in an urban area has not been presented. In this paper, we used corner feature information for precise vehicle localization in an urban area.

In indoor environments, corridor walls can be clearly scanned by 2D LIDAR [18,19,20,21]. This is because there is almost no object covering the corridor wall. Therefore, the corner can be easily extracted by 2D LIDAR. However, in urban environments, corner extraction using only 2D LIDAR is difficult. Because of the many roadside trees in urban areas, LIDAR can scan only a part of the building. Also, if the outer wall of the building is made of glass, the laser cannot be reflected. In this case, by using 3D LIDAR, the false positive rate can be reduced through combining the multiple corner extraction results from each layer. The corner map contains information describing the geometrical properties of the vertical corner of the corresponding building. The vehicle position error can be corrected through corner map matching. This corner map has the advantage of having a small data file size.

The Extended Kalman Filter (EKF) is used to estimate the vehicle position. The vehicle motion is updated using the output from the ICP algorithm based on the geometric relations between the scan data of 3D LIDAR. The vertical corner is extracted using the proposed corner extraction method. The vehicle position is then corrected by matching the prebuilt corner map with the extracted corner. The experiment was carried out in the Gangnam area of Seoul, South Korea.

The remaining part of this paper is organized as follows. In Section 2, the vertical corner feature is defined and the corner extraction method is proposed. The procedure for the generation of the vertical corner map is described in Section 3. In Section 4, the Kalman filter configuration and the observability analysis are described. The vehicle driving test results are shown in Section 5. Finally, the conclusion is given in Section 6.

## 2. Corner Extraction

In general, a corner is a position formed by the interconnection of two converging lines or surfaces. The outer wall of the building is almost flat, forming a vertical plane perpendicular to the ground. Therefore, the vertical corner could be a distinctive and useful point landmark for the map based vehicle localization in an urban area. In general, each building has at least four vertical corners and these can be extracted using LIDAR.

### 2.1. Corner Definition

**Figure 1:**
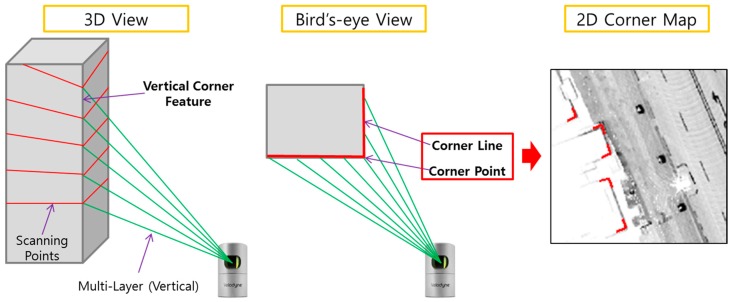
Corner definition.

In this paper, the vertical corner is projected into the ground plane and treated as a point feature on the 2D horizontal plane. Figure 1 shows the corner definition.

**Figure 2:**
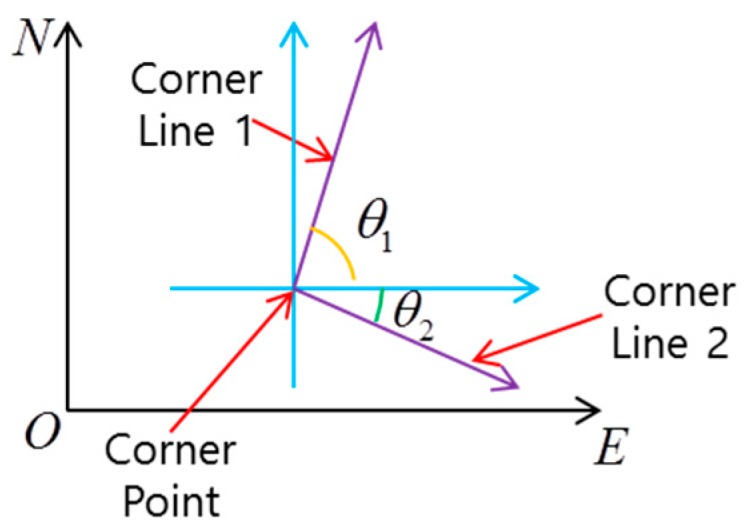
Attribute of the corner feature.

As shown in Figure 1, the corner consisted of a corner point and two corner lines. Figure 2 shows the attributes of the corner.

The corner point is represented by a 2D horizontal position in the east-north-up (ENU) frame. The corner line has a directional property of the corresponding building. Therefore, as shown in Figure 2, the corner line is represented by a directional angle ($\theta_{1}$, $\theta_{2}$) in the ENU frame.

### 2.2. Corner Extraction

In this paper, the vehicle localization utilizes the vertical corner feature of the building. Therefore, we only use the data of the eight upper layers of the 3D LIDAR [22]. The scanned data is processed for each layer.

#### 2.2.1. Line Extraction

**Figure 3:**
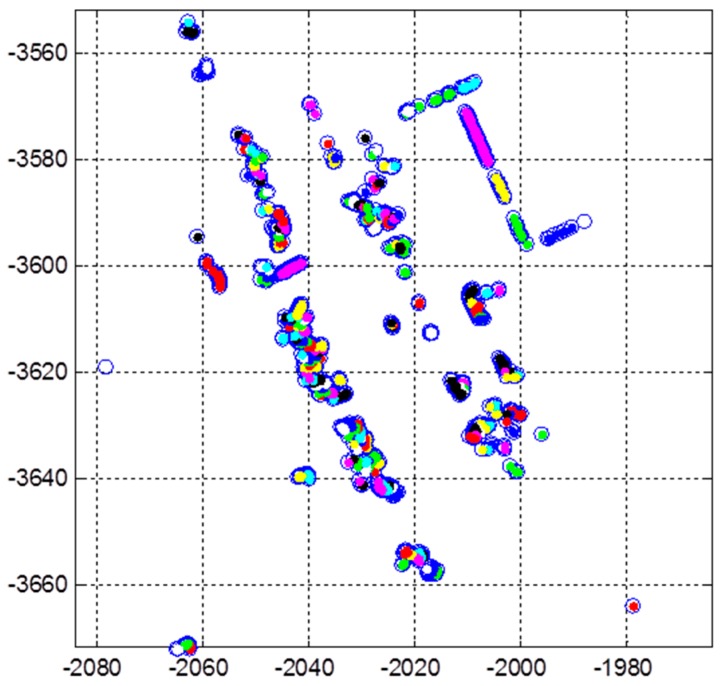
Line extraction result using the IEPF algorithm.

In order to extract the corner, the line must be accurately extracted. Several line extraction methods have previously been presented [23,24,25,26]. In these methods, the Iterative-End-Point-Fit (IEPF) algorithm shows the best performance in terms of accuracy and computational time [27]. Figure 3 shows the line extraction result using the IEPF algorithm.

As shown in Figure 3, the laser scanning points are divided into many line segments.

#### 2.2.2. Outlier Removal

**Figure 4:**
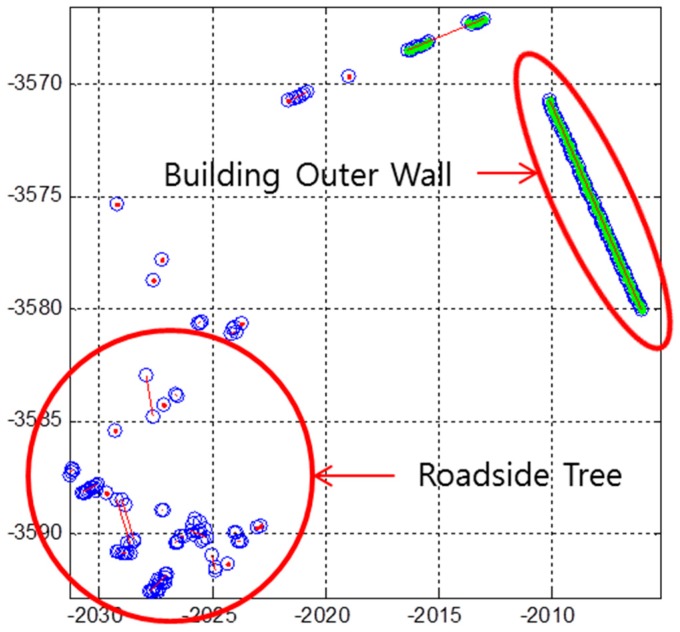
Laser scanning points reflected by the leaves of roadside trees and the outer wall of the building.

In Figure 3, most of these line segments are a set of scan points from the leaves of the roadside trees. Therefore, the laser data reflected by the leaves of roadside trees must be removed. Figure 4 shows the laser scanning points reflected by the leaves and the outer wall of the building.

**Figure 5:**
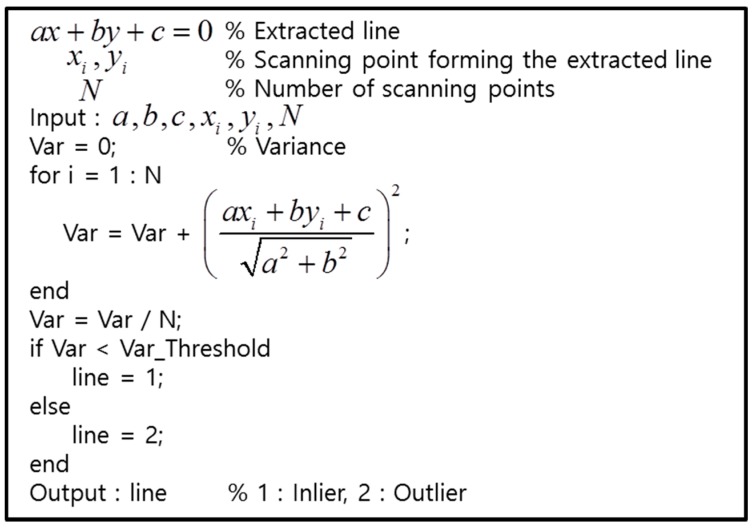
Pseudo code for outlier removal.

As shown in Figure 4, the features of the laser scanning points that are reflected by the two types of objects are clearly distinguished. For roadside trees, the variance of distance errors between the extracted line and each point is very large. On the other hand, for outer wall of a building, the variance of the distance errors is very small. Figure 5 shows the pseudo code for outlier removal.

**Figure 6:**
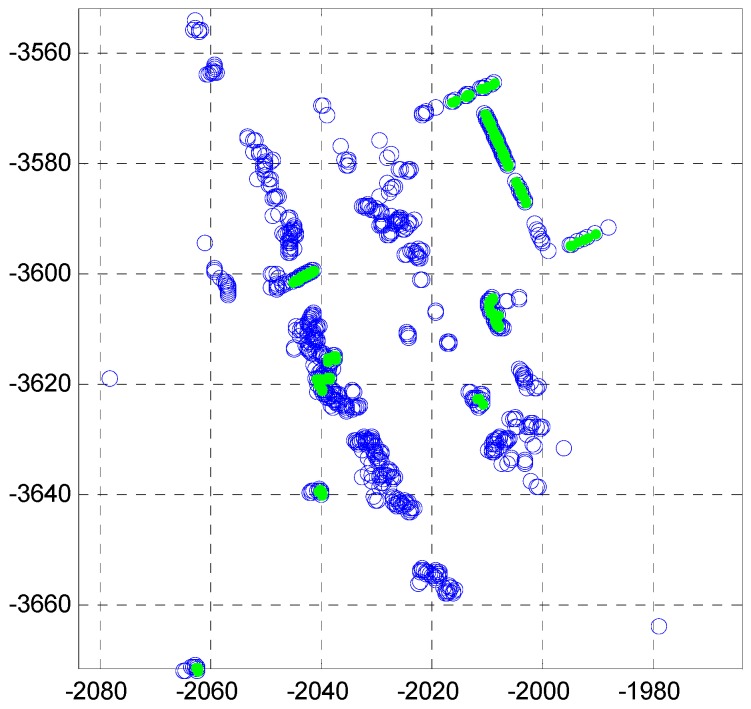
Line extraction result (after outlier removal).

Through the process of the pseudo code, the outliers such as the roadside trees are removed. Figure 6 shows the line extraction result after the outlier removal.

In Figure 6, the green points represent the line segments that are finally extracted.

#### 2.2.3. Corner Candidate Extraction

**Figure 7:**
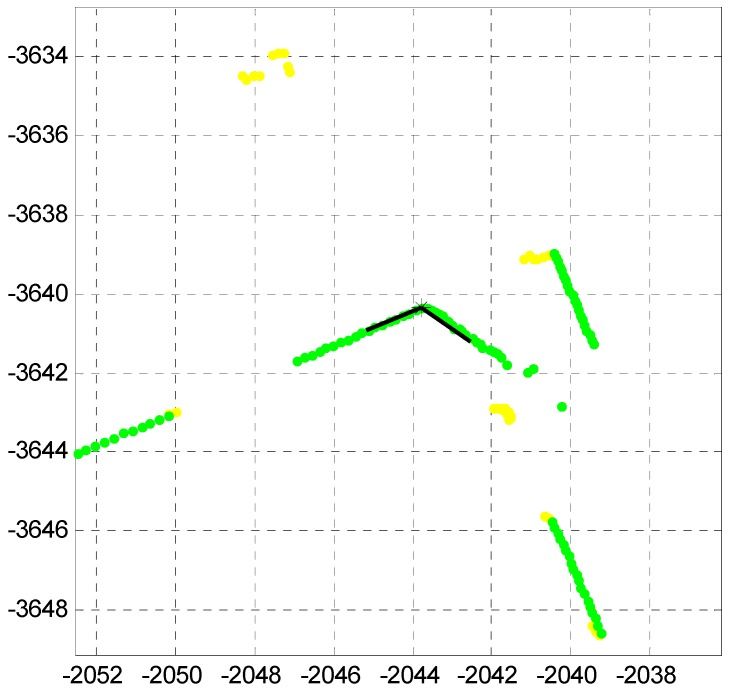
Extracted corner candidate (black star and lines).

By the corner definition of the Section 2.1, the corner candidate is extracted. Figure 7 shows the extracted corner candidate.

As shown in Figure 7, the corner point (black star) and the corner lines (black lines) are extracted as the corner candidate.

#### 2.2.4. Corner Determination

**Figure 8:**
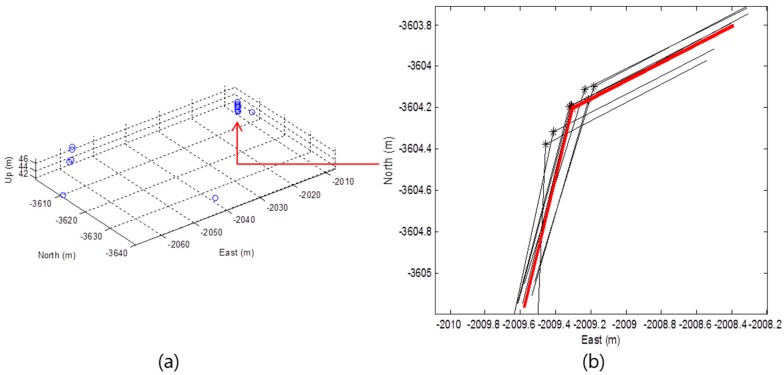
(**a**) 3D position of corner candidates; (**b**) Corner determination result.

By using 3D LIDAR, the false positive rate can be reduced through the redundant corner candidates from each layer. Figure 8 shows the 3D position of the corner candidates extracted from the eight layers and the corner determination result.

In Figure 8a, the multiple corner candidates from each layer can be found. Generally, the outer wall of the building is erected vertically to the ground. Thus, as shown In Figure 8b, the 2D positions of the corner candidates extracted in one vertical corner are approximately the same. The corner candidates that exist within a certain area in the 2D east-north frame are classified into the same corner. Then, if the number of corner candidates that exist within a certain area is greater than a predetermined number (redundancy check), the corner is determined. In Figure 8b, the position and angle of the determined corner (red) is the average value of the corner candidates (black) for the corresponding corner.

#### 2.2.5. Corner Extraction Result

**Figure 9:**
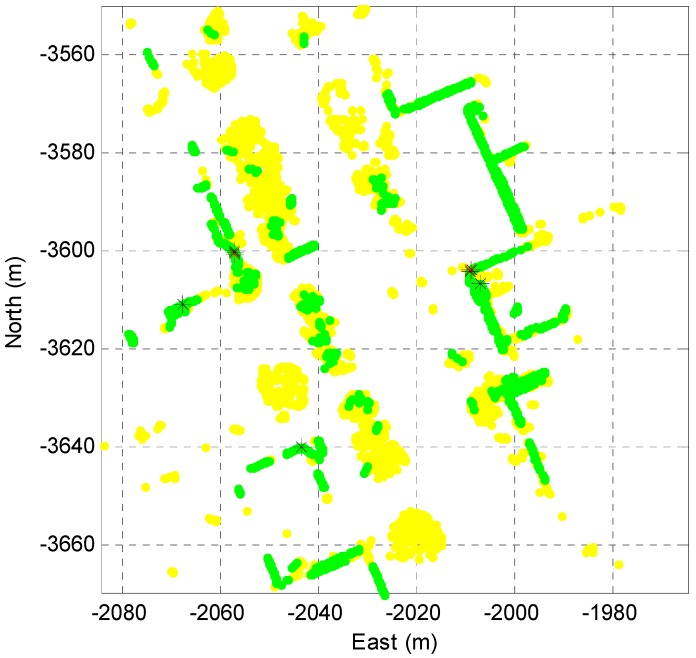
Corner extraction result.

Figure 9 shows the final corner extraction result.

In Figure 9, the extracted line segments are represented in green and the objects (outlier) such as the roadside trees are represented in yellow. The stars represent the corner candidates and the red stars represent the final extracted corners. As shown in Figure 8 and Figure 9, all of the black stars were extracted from only one layer and these were excluded from the redundancy check.

## 3. Corner Map Generation

A corner map is essential for precise vehicle localization. By using the corner map, the vehicle position can be very accurately estimated.

### 3.1. Corner Map Definition

**Figure 10:**
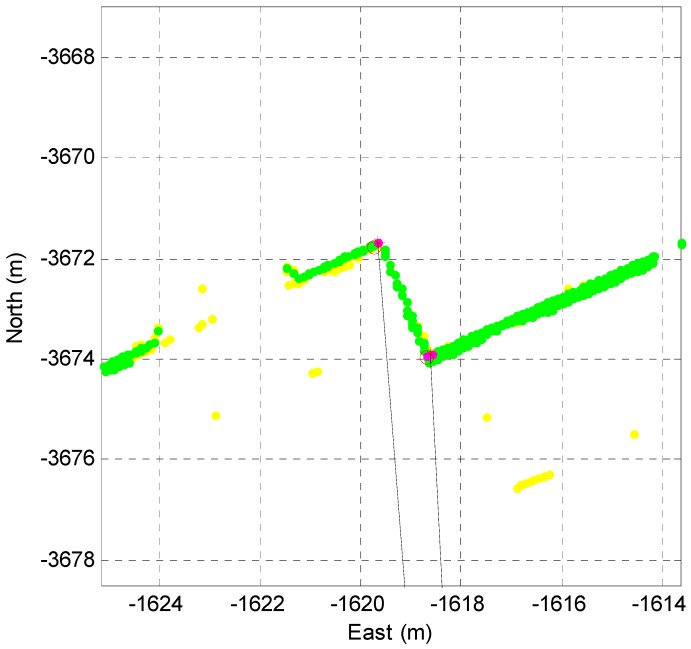
Extraction result of the two nearest corners.

Basically, a corner map must have the 2D positions of the corners. However, if the corner map only has the position information of the corners, problems can arise in the data association process. Figure 10 shows an extraction result of the two nearest corners.

In Figure 10, the red circles represent the position of the corner map. Also, the red stars represent the extracted corners, where the distance between the two corners is approximately 2.5 m. In this situation, when the position error of the vehicle has occurred more than 1 m from the true position, the possibility of data association failure greatly increased. Therefore, the direction angle information of the two corner lines is added to the corner map (see Figure 2). The two corners shown in Figure 10 can be clearly identified by using the direction angle information of the corner lines.

Finally, to determine the quality of the extracted corner information, the position error covariance of the corner is added to the corner map. Table 1 shows an example of a corner map.

As shown in Table 1, the corner map information is saved as a text file. Based on a driving path of about 2 km, the number of extracted corners is 271 and the text file size is 28 KB. The corner map therefore has the advantage of being able to cover a large area with a very small data file size.

### 3.2. Corner Map Generation

**Figure 11:**
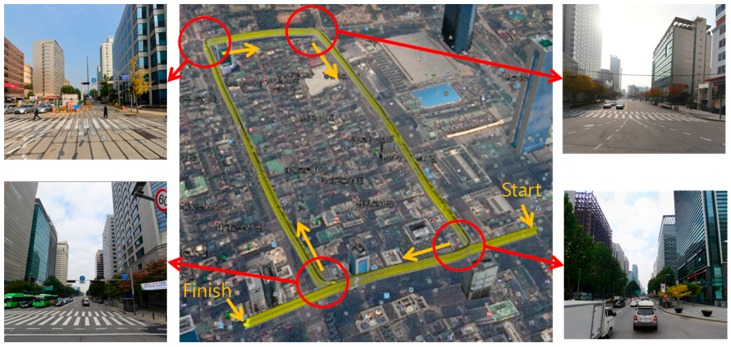
Vehicle trajectory and street view (four intersections).

The experiment has been carried out in the Gangnam area of Seoul, South Korea. Figure 11 shows the vehicle trajectory and the street view at the four intersections.

**Figure 12:**
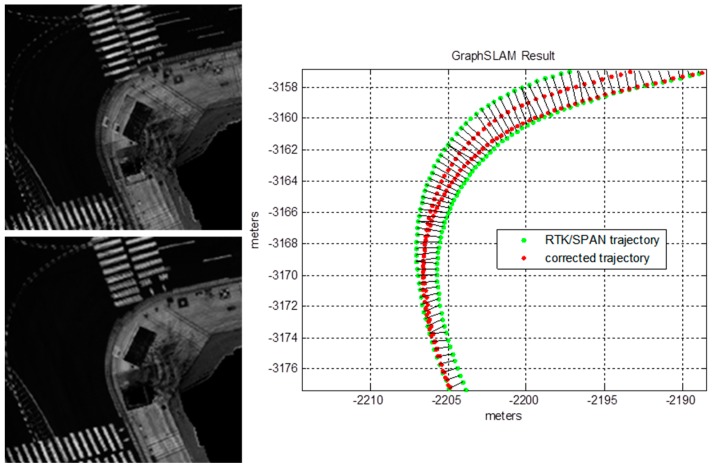
Graph optimization result of the vehicle trajectory using GraphSLAM.

As shown in Figure 11, the experimental environment is a dense urban area surrounded by high buildings, and two laps were driven from the starting point to the finish point. The traveling distance was about 4.5 km and the maximum traveling speed was about 40 km/h. The position and heading of the vehicle were acquired by using the integrated system of the Real-Time Kinematic (RTK) GPS and Inertial Navigation System (INS) (NovAtel RTK/SPAN system). In an environment where there are many high buildings, the position error of the RTK/INS is about 1–2 m. Therefore, the vehicle trajectory must be corrected to generate the corner map. The vehicle trajectory was optimized by using the GraphSLAM method [28,29]. In this paper, this optimized vehicle trajectory was considered as the ground truth. Figure 12 shows the graph optimization result of the vehicle trajectory.

As shown at the top left of Figure 12, the intensity maps for the two laps do not match. On the right of Figure 12, the red points represent the corrected vehicle trajectory after the graph optimization. As shown at the bottom left of Figure 12, the intensity map exactly matches. Here, the incremental pose information outputted from the ICP algorithm was used as edge measurement of the graph. The theory and principles for the GraphSLAM method are well described in [28,29]. Thus, obtaining the optimized vehicle trajectory is possible by using the GraphSLAM.

**Figure 13:**
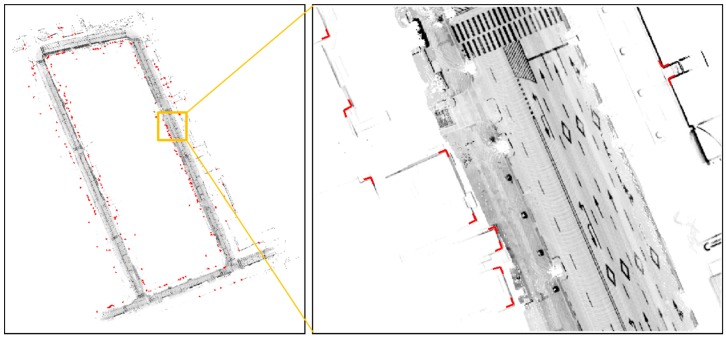
Generated corner map information (on synthetic map).

The corner map is generated based on the ground truth. In addition, by applying the corner extraction method described in Section 2, the data of the corner extracted from each vehicle position are collected. In the next, clustering is performed to classify the data in such a corner. Also, the mean position, mean direction angle, and position error covariance of the corner for each cluster are calculated. Finally, to increase the reliability of the corner map, the corners with a large position error covariance and a small extracted count are excluded from the corner map. Figure 13 shows the final generated corner map information displayed on the synthetic map of the 2D occupancy grid and road intensity maps.

As shown in Figure 13, many corners were extracted, and false positives did not occur.

### 3.3. Data Association with the Corner Map

**Figure 14:**
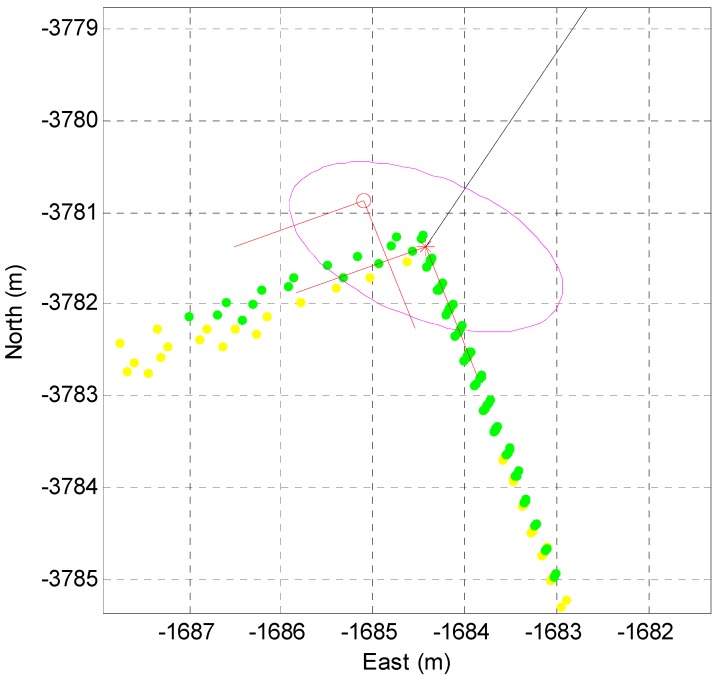
Data association with the corner map.

First, the extracted corners need to be associated with the corner map data. The data association considers the position error covariance of the vehicle. Figure 14 shows the data association process between the extracted corners and the corner map.

In Figure 14, the red circle indicates the position of the corner map and the red star shows the position of the extracted corner. The red lines represent the directional angle of the corner lines, and the magenta ellipse represents the position uncertainty of the corner caused by the position error covariance of the vehicle. As shown in Figure 14, when the red circle appears in the elliptic region and the difference between the directional angles of the map and the extracted corner is less than a predetermined angle value, the two corners are determined to be the same corner.

## 4. Kalman Filter Configuration and Observability Analysis

In this paper, the general 2D point landmark-based positioning technique is used to estimate the vehicle position [30]. The landmarks are the vertical corners of the building in the street.

### 4.1. Motion Update

The motion update process is predicted with the translation vector (T) and the rotation matrix (R) from the ICP algorithm based on the range data of 3D LIDAR. The state variables are defined as follows.

(1) $$\begin{array}{l} q={\left(x\;,\;y\;,\;\theta \right)}^{T}\\ u={\left(T\;,\;R\right)}^{T} \end{array}$$

where x and y are the horizontal (2D) positions of the vehicle in the ENU frame, and θ is the heading angle of the vehicle. T and R refer to the increment of the 2D position and the heading angle, respectively. The state equation is as follows.

(2) $$\begin{array}{l} q_{k+1}=F_{K}q_{k}+G_{k}u_{k}\\ F_{k}=\left[\begin{array}{ccc} 1 & 0 & 0\\ 0 & 1 & 0\\ 0 & 0 & 1 \end{array}\right]\;G_{k}=\left[\begin{array}{ccc} \cos\left(\theta_{k}\right) & -\sin\left(\theta_{k}\right) & 0\\ \sin\left(\theta_{k}\right) & \cos\left(\theta_{k}\right) & 0\\ 0 & 0 & 1 \end{array}\right] \end{array}$$

In Equation (2), the vehicle motion is updated by accumulating the results of the ICP.

### 4.2. Measurement Update

The range and bearing measurements between the vehicle and the corners are used for the measurement update. The measurement equations are as follows.

(3) $$r_{i}=\sqrt{{\left(x_{i}-x\right)}^{2}+{\left(y_{i}-y\right)}^{2}}$$

(4) $$\alpha_{i}=\tan^{-1}\left(\frac{y_{i}-y}{x_{i}-x}\right)-\theta$$

where $r_{i}$ and $\alpha_{i}$ are the range and bearing measurements between the vehicle and the *i*th corner, respectively. $x_{i}$ and $y_{i}$ are the 2D position of the *i*th corner in the corner map. The measurement matrix H is as follows.

(5) $$H=\left[\begin{array}{ccc} \frac{-\left(x_{1}-x\right)}{\sqrt{{\left(x_{1}-x\right)}^{2}+{\left(y_{1}-y\right)}^{2}}} & \frac{-\left(y_{1}-y\right)}{\sqrt{{\left(x_{1}-x\right)}^{2}+{\left(y_{1}-y\right)}^{2}}} & 0\\ \frac{\left(y_{1}-y\right)}{{\left(x_{1}-x\right)}^{2}+{\left(y_{1}-y\right)}^{2}} & \frac{-\left(x_{1}-x\right)}{{\left(x_{1}-x\right)}^{2}+{\left(y_{1}-y\right)}^{2}} & -1\\ \vdots & \vdots & \ddots \\ \frac{-\left(x_{n}-x\right)}{\sqrt{{\left(x_{n}-x\right)}^{2}+{\left(y_{n}-y\right)}^{2}}} & \frac{-\left(y_{n}-y\right)}{\sqrt{{\left(x_{n}-x\right)}^{2}+{\left(y_{n}-y\right)}^{2}}} & 0\\ \frac{\left(y_{n}-y\right)}{{\left(x_{n}-x\right)}^{2}+{\left(y_{n}-y\right)}^{2}} & \frac{-\left(x_{n}-x\right)}{{\left(x_{n}-x\right)}^{2}+{\left(y_{n}-y\right)}^{2}} & -1 \end{array}\right]$$

### 4.3. Observability Analysis

The observability of the vehicle state estimation using the corner point measurement is required to be verified. This observability of the vehicle state is analyzed in the algebraic framework [31]. First, the state evolution model is as follows.

(6) $$\begin{array}{l} \dot{x}=\left\|T\right\|\cdot \cos\theta \\ \dot{y}=\left\|T\right\|\cdot \sin\theta \\ \dot{\theta }=R \end{array}$$

where ‖⋅‖ is the Euclidean norm and R is a 1 × 1 matrix. Expressing Equation (4) with respect to θ is as follows.

(7) $$\theta =\tan^{-1}\left(\frac{y_{i}-y}{x_{i}-x}\right)-\alpha_{i}$$

By taking the derivative with respect to time of Equation (7), we obtain Equation (8).

(8) $$\dot{\theta }=\frac{1}{1+{\left(\frac{y_{i}-y}{x_{i}-x}\right)}^{2}}\left\{\dot{x}\cdot \frac{y_{i}-y}{{\left(x_{i}-x\right)}^{2}}-\dot{y}\cdot \frac{1}{x_{i}-x}\right\}$$

By substituting Equation (6) in Equation (8), we obtain the following equation.

(9) $$R=\frac{1}{1+{\left(\frac{y_{i}-y}{x_{i}-x}\right)}^{2}}\left\{\left\|T\right\|\cdot \cos\theta \cdot \frac{y_{i}-y}{{\left(x_{i}-x\right)}^{2}}-\left\|T\right\|\cdot \sin\theta \cdot \frac{1}{x_{i}-x}\right\}$$

By substituting Equation (7) in Equation (9), we obtain Equation (10).

(10) $$R=\frac{\left\|T\right\|\cdot \left[\frac{y_{i}-y}{x_{i}-x}\cdot \cos\left\{\tan^{-1}\left(\frac{y_{i}-y}{x_{i}-x}\right)-\alpha_{i}\right\}-\sin\left\{\tan^{-1}\left(\frac{y_{i}-y}{x_{i}-x}\right)-\alpha_{i}\right\}\right]}{\left(x_{i}-x\right)\left\{1+{\left(\frac{y_{i}-y}{x_{i}-x}\right)}^{2}\right\}}$$

In Equations (3) and (10), $r_{i}$ is the range measurement, T and R are the input values from the ICP, $x_{i}$ and $y_{i}$ represent the position information from the corner map, and x and y are unknowns. Since there are two equations and two unknown variables, it is possible to calculate x and y. Therefore, x and y are observable. In Equation (7), since $\alpha_{i}$ is a measurement, θ is also observable.

From this observability analysis, if at least more than one corner feature can be extracted while the vehicle is moving, the vehicle position can be estimated from the corner map matching.

## 5. Experimental Results

In this section, we analyze the experimental results by comparing the corner map matching, RTK/INS, and ICP based Dead Reckoning (DR). We also analyze the causes of the position error when the corner map matching is used.

**Figure 15:**
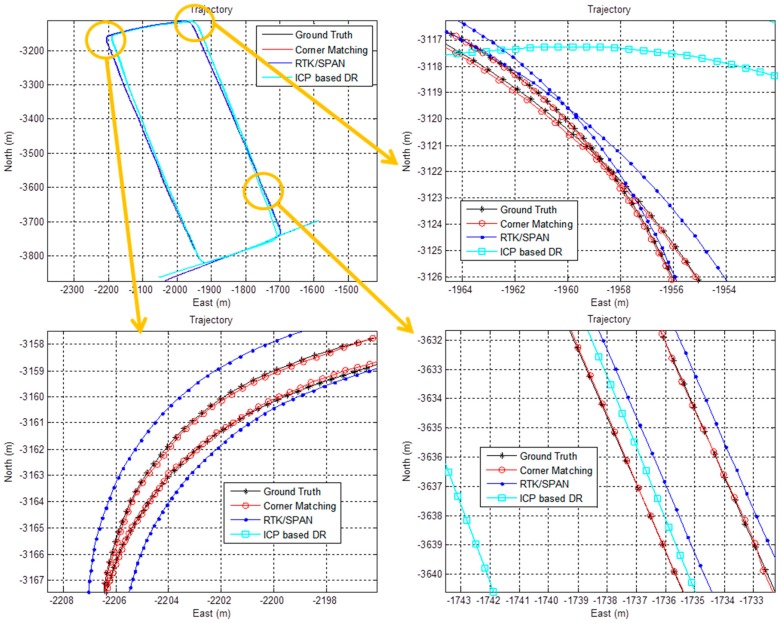
Vehicle trajectory for each case.

**Figure 16:**
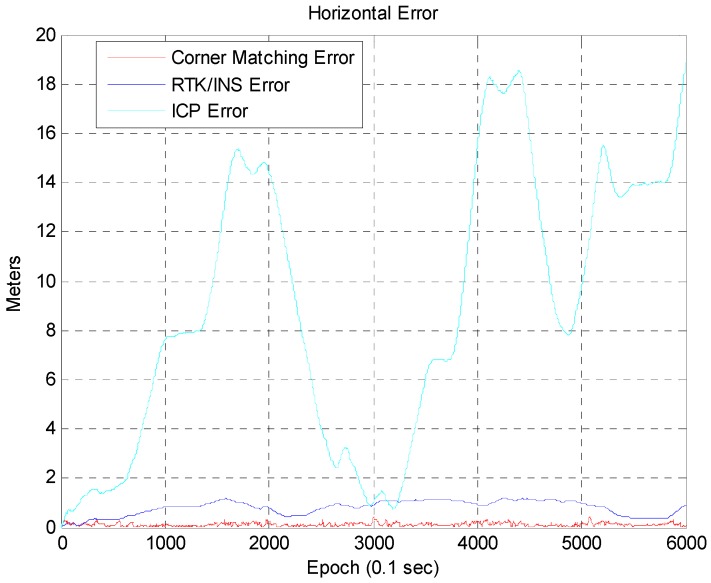
Position error (using only the ICP based DR).

**Figure 17:**
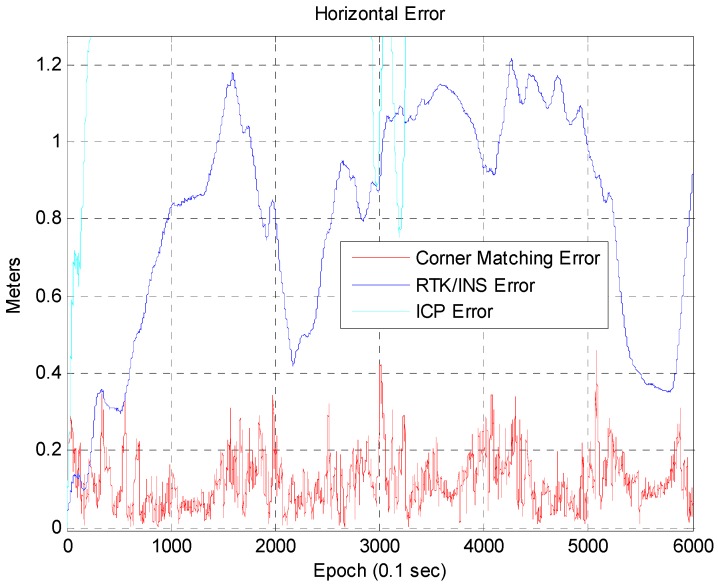
Position error (corner map matching and RTK/INS).

Figure 15 shows the vehicle trajectory for each case. The trajectory for the corner map matching is almost the same as the ground truth. In the case of RTK/INS, the trajectory has a small offset error with respect to the ground truth. The trajectory for the ICP based DR significantly differs from the ground truth. Figure 16 shows the accumulated position error that occurs when using only the ICP based DR without the corner map matching. As is well known, the position error of the ICP based DR is divergent. This accumulated position error can be removed by using the corner map matching. Figure 17 shows the result of the corner map matching.

**Figure 18:**
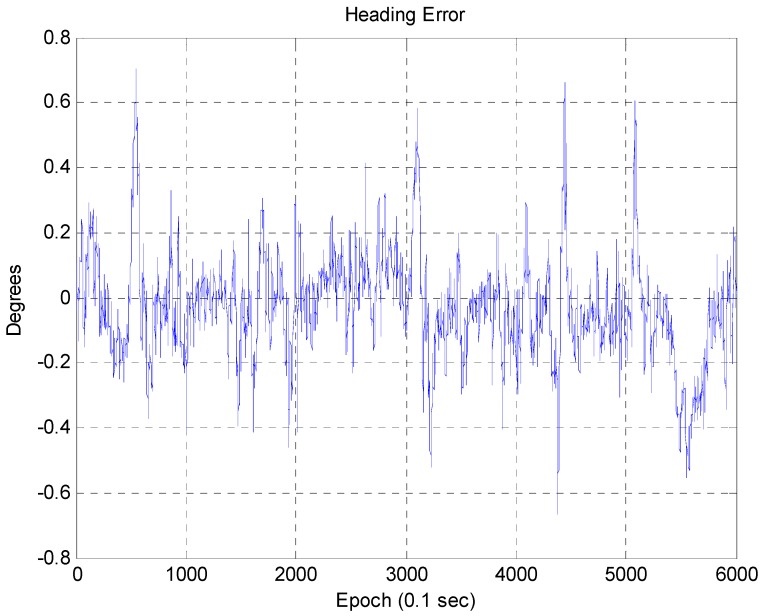
Heading error (corner map matching).

**Figure 19:**
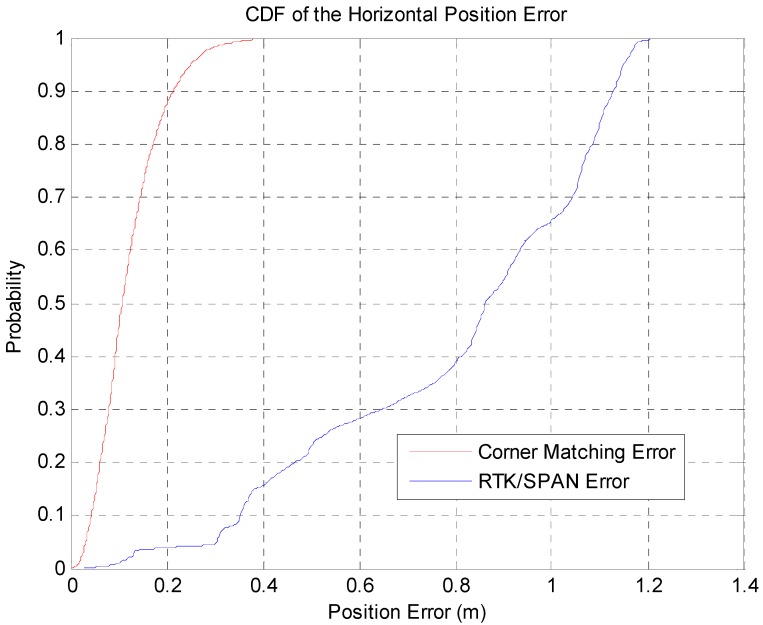
CDF of the horizontal vehicle position error.

In Figure 17, the blue line represents the position error of the RTK/INS, and the red line represents the position error of the corner map matching. The maximum position error of the RTK/INS is about 1.2 m. Here, the position error of the RTK/INS at the start point is very small because the vehicle trajectory is optimized based on the start point. On the other hand, the maximum position error of the corner map matching is about 0.46 m. The 2D RMS horizontal errors of the RTK/INS and the corner map matching are about 0.85 m and 0.138 m, respectively. In Figure 18, it can be seen that the heading error is very small. The RMS heading error of the corner map matching is about 0.168°. Figure 19 shows the Cumulative Distribution Function (CDF) of the horizontal vehicle position error.

**Figure 20:**
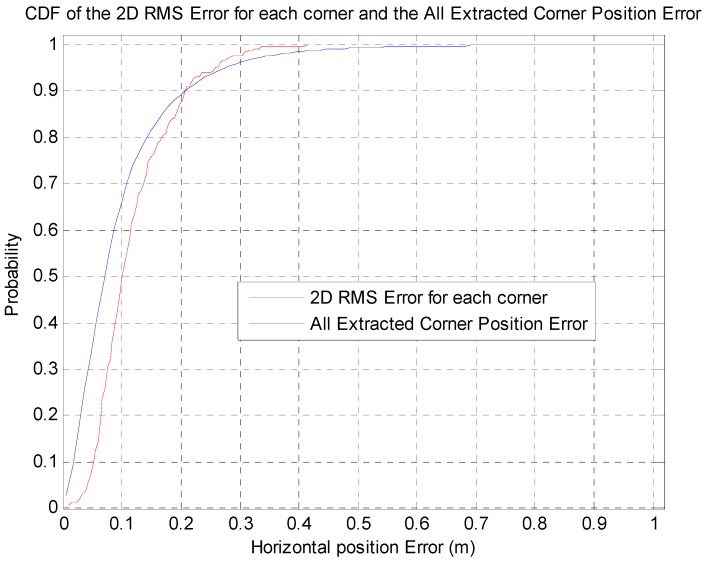
CDF of the 2D RMS horizontal errors for each corner and the horizontal position errors of all extracted corners.

The corner map matching has localization accuracies of about 0.25 m and 0.33 m at 95% and 99% confidence levels, respectively. Figure 20 shows the CDF of the 2D RMS horizontal errors for each corner (271 corners) and the horizontal position errors of all the extracted corners (13,260 corner extraction data).

In Figure 20, the horizontal position accuracies of all the extracted corners are about 0.27 m and 0.45 m at 95% and 99% confidence levels, respectively. Each corner has 2D RMS horizontal errors of about 0.26 m and 0.32 m at 95% and 99% confidence levels, respectively. As shown in Figure 19 and Figure 20, the corner map matching error is very small, since the corner measurement is accurate. Also, the CDF of the 2D RMS error for each corner shows that the position error for each corner is affected by the characteristic of the corresponding corner.

**Figure 21:**
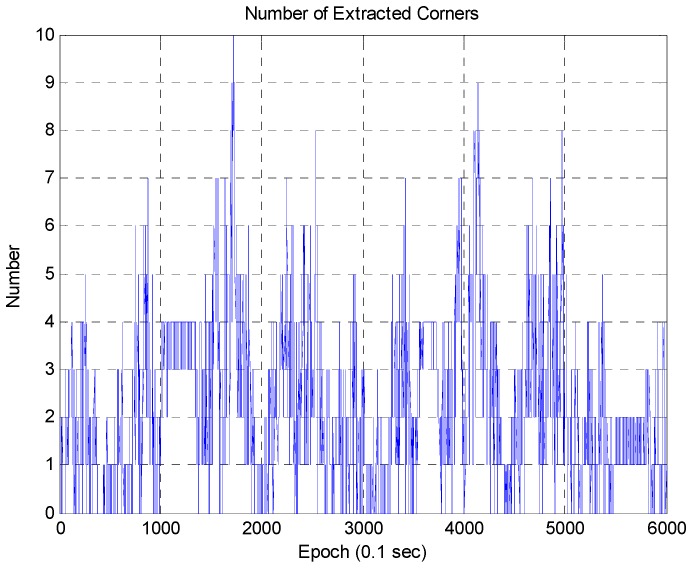
Number of extracted corners.

As shown in Figure 17 and Figure 19, the corner map matching errors are greater than 0.33 m at several regions. These errors are related to the number of extracted corners, distance from the vehicle to the corner, and corner shape. First, let us consider the number of extracted corners as shown in Figure 21.

**Figure 22:**
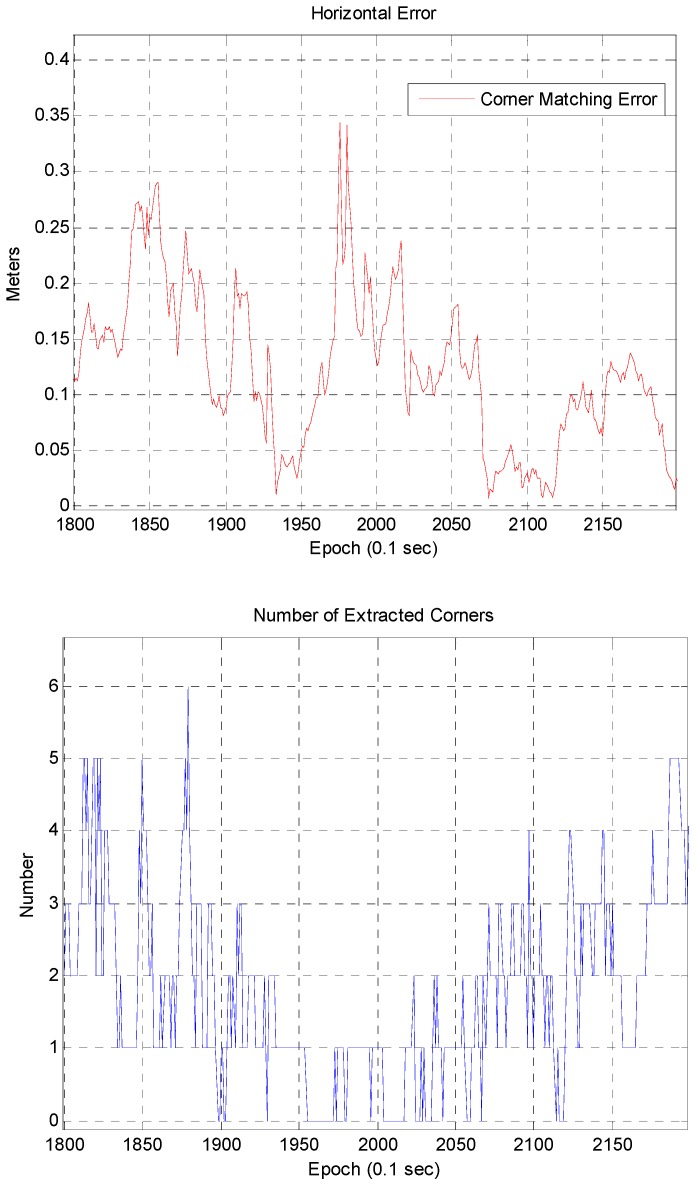
Relationship between the localization error and the number of extracted corners.

**Figure 23:**
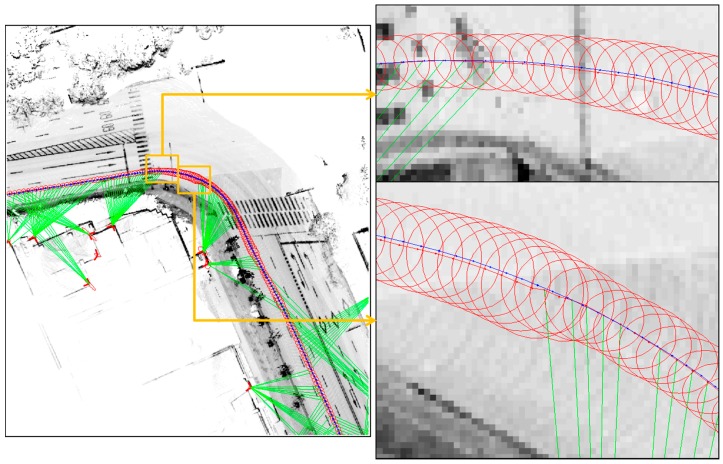
Vehicle trajectory with the position error covariance (the blue trajectory is the ground truth; the red trajectory is the estimated trajectory; the red ellipses represent the vehicle position error covariance; the green lines are to connect between the extracted corners and the vehicle position).

Figure 21 shows that there are less than five extracted corners in most areas. Since there are many roadside trees in urban areas, it is impossible to extract a large number of corners. Furthermore, if the building has glass walls, the corner cannot be extracted. Figure 22 shows the relationship between the localization error and the number of extracted corners. On the left of Figure 22, the localization error largely occurs in the epoch from 1950 to 2020. On the right of Figure 22, it can be seen that the corner was barely extracted at the same epoch. When the corner is not extracted, the position error of the ICP based DR is accumulated. However, as shown in Figure 22, the accumulated position error is reduced at the same time as a corner is extracted. Figure 23 shows the vehicle trajectory with the position error covariance at the same epoch.

At the bottom right of Figure 23, when the corner was extracted, the accumulated position error and the position error covariance were reduced. Here, the ellipse of the covariance is not of actual size, because it was expanded for clarity. In this way, even by extracting only one corner, the vehicle state can be estimated as demonstrated in Section 4.3. Of course, if more corners are extracted, the localization performance can be improved.

**Figure 24:**
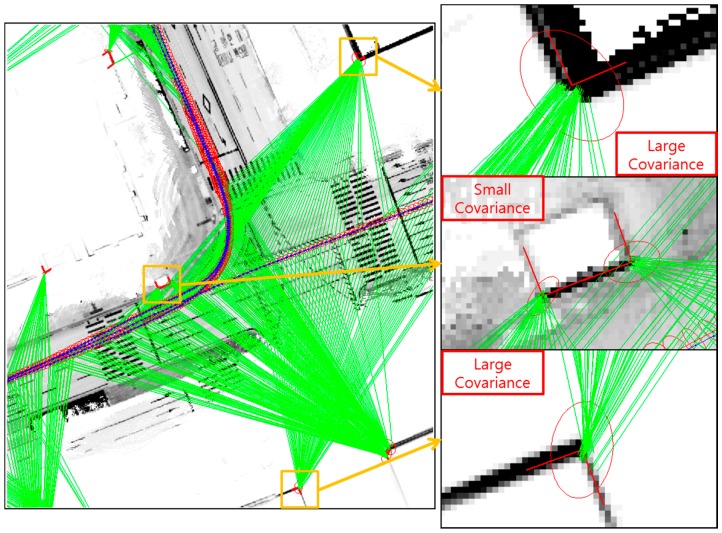
Relationship between the corner position error covariance and the distance from the vehicle to the corner (the red ellipses on the corner represent the position error covariance of the extracted corners).

Secondly, the localization accuracy is related to the distance from the vehicle to the corner. Figure 24 shows the relationship between the corner position error covariance and the distance from the vehicle to the corner.

**Figure 25:**
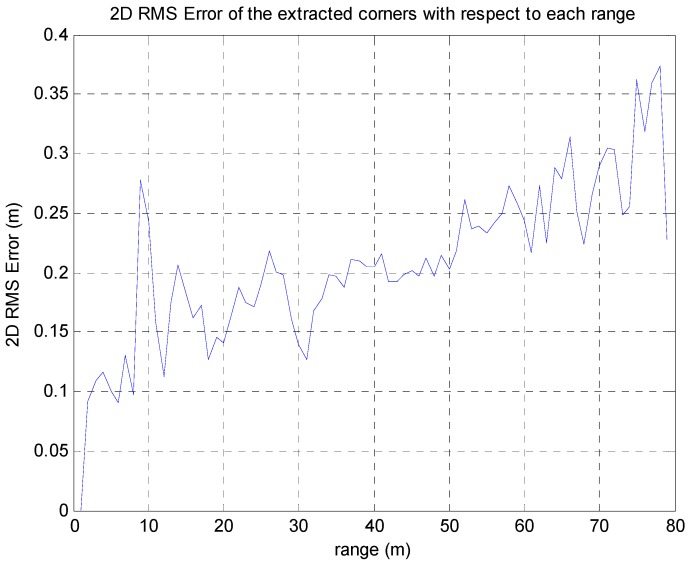
2D RMS error of the extracted corners with respect to each distance.

As shown in Figure 24, the position accuracy of the extracted corner is most closely related to the distance from the vehicle to the extracted corner. The further the corner is from the vehicle, the greater is the error covariance of the corner position. This is because, as the distance to the object increases, the more the ranging accuracy of the LIDAR is reduced. Also, the horizontal resolution of the laser beam is 0.16°. If any object is 50 m from the vehicle, the position uncertainty of the reflected point is 0.14 m. Figure 25 shows that the 2D RMS error of the extracted corners increases in proportion to the distance. Thus, the measurement of the corner that is furthest from the vehicle could be the cause of the relatively large error.

**Figure 26:**
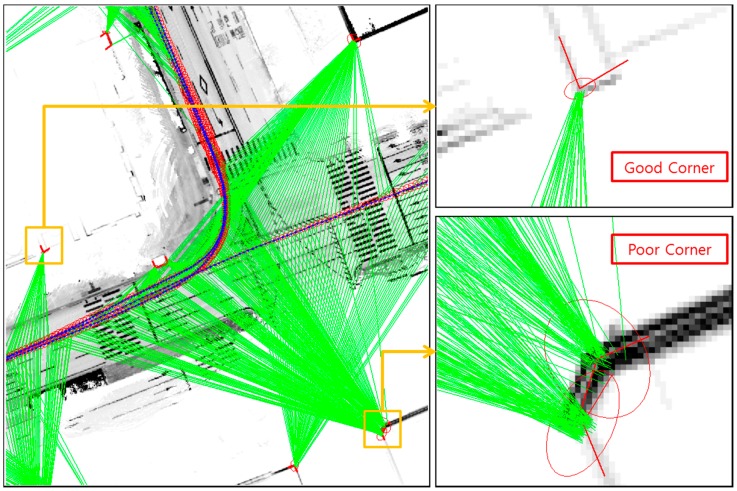
Examples of good and poor corners for the corner shape (the red ellipses on the corner represent the position error covariance of the extracted corners).

Finally, let us consider the corner shape. Figure 26 shows the examples of good and poor corners for the corner shape.

**Figure 27:**
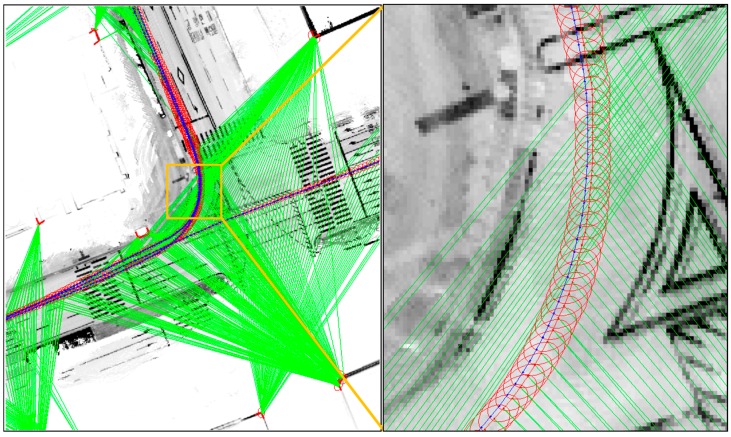
Estimated vehicle trajectory in the situation affected by the poor corner measurement (the blue trajectory is the ground truth).

In the good corner example, the angle between the two corner lines is perpendicular, and the error covariance is very small. The position accuracy of the extracted corner is related to the Dilution of Precision (DOP) between the two corner lines [32]. When the angle between the two corner lines is perpendicular, the DOP of the corner position is minimized. However, as shown at the bottom right of Figure 26, the position of the poor corner has a large uncertainty. Figure 27 shows the estimated vehicle trajectory in a situation affected by the poor corner measurement with a large covariance.

As shown on the right of Figure 27, the corner map matching error caused by the poor corner measurement is relatively large. These errors are about 0.3 m.

So far, the vertical corner feature based precise vehicle localization has been described. The results show that the corner map matching performance is determined by the accuracy of the extracted corner. The corner is a very good landmark which can be extracted accurately. Therefore, the vehicle position can be estimated accurately. The results also show that the corner map matching method can guarantee very good localization accuracy in an urban area. The performance can be further improved by accurately extracting more corners.

## 6. Conclusions

In this paper, we proposed the vertical corner feature based precise vehicle localization method using 3D LIDAR in an urban area. First, we defined a corner map which was generated using the proposed corner extraction method as described in Section 3. After generating the corner map, we estimated the vehicle position using corner map matching. In the experimental results, the maximum and 2D RMS horizontal errors generated by using the corner map matching were about 0.46 m and about 0.138 m, respectively.

The vertical corner feature based precise vehicle localization method has several advantages. First, the corner map matching performance is very good and 2D RMS horizontal error is about 0.138 m. Second, in contrast to the road intensity map based localization methods, the corner map matching method can be used in a condition of traffic congestion. Third, the data file size of the corner map is very small compared to that of the road intensity map. The calculation time of the corner map matching is also very short compared to the other map matching methods.

As previously mentioned, the accuracy of the corner measurements and the number of extracted corners are very important for the precise vehicle localization. Accordingly, a method for extracting many corners with higher accuracy should be investigated in the future.

## Figures and Tables

**Table 1:** An example of a corner map.

Index	East (m)	North (m)	Direction Angle 1 (Degree)	Direction Angle 2 (Degree)	Covariance Matrix (2 × 2)			
1	10	10	132	45	0.0021	0.0009	0.0009	0.0029
2	20	30	−13	−13	0.0042	−0.001	−0.001	0.0015
